# Establishment and Validation of a Preoperative MRI-based Nomogram for Predicting the Risk of Malignancy in Patients with Breast Tumor

**DOI:** 10.7150/jca.49441

**Published:** 2021-01-01

**Authors:** Jianguo Lai, Jinjiang Lin, Hongli Wang, Yi Sun, Yudong Li, Huan Tian, Shiyu Shen, Cui Tan, Huanhuan Liu, Fengyan Yu

**Affiliations:** 1 Department of Breast Surgery, Breast Tumor Center, Sun Yat-Sen Memorial Hospital, Sun Yat-Sen University, Guangzhou, Guangdong, China.; 2 Department of Radiology, The First Affiliated Hospital of Sun Yat-Sen University, Guangzhou, Guangdong, China.; 3 Diagnostic Department, Breast Tumor Center, Sun Yat-Sen Memorial Hospital, Sun Yat-Sen University, Guangzhou, Guangdong, China.; 4 Guangdong Provincial Key Laboratory of Malignant Tumor Epigenetics and Gene Regulation, Sun Yat-Sen Memorial Hospital, Sun Yat-Sen University, Guangzhou, Guangdong, China.; 5 Department of Pathology, Sun Yat-Sen Memorial Hospital, Sun Yat-Sen University, Guangzhou, Guangdong, China.; 6 Department of Breast Cancer, Cancer Center, Guangdong Provincial People's Hospital and Guangdong Academy of Medical Science, Guangzhou, Guangdong, China.; 7 Department of Plastic Surgery, Sun Yat-sen Memorial Hospital, Sun Yat-sen University, Guangzhou, Guangdong, China.

**Keywords:** Breast, Nomogram, Magnetic resonance imaging, Diagnosis

## Abstract

**Purpose:** To establish a preoperative nomogram incorporating morphological and dynamic contrast-enhanced (DCE) features to individually predict the risk of malignancy in patients with breast tumor.

**Methods** A total of 447 consecutive female patients who were divided into the primary cohort (n=326) and the validation cohort (n=121) were enrolled between March 2015 to January 2018. Univariate and multivariate logistic regression analyses were used to identify the potential independent indicators of malignancy. An MRI-based nomogram integrating morphological features and kinetic curves was developed to achieve individualized risk prediction of malignancy in patients with breast masses. The discrimination, calibration ability and clinical utility of the MRI-based model were assessed using C-index, calibration curve and decision curve analysis.

**Results:** Age, tumor size, margin, internal enhancement characteristics, and kinetic curve were confirmed as the independent predictors of malignancy. The AUC of MRI-based nomogram was 0.940 (95% CI: 0.911-0.970) and 0.894 (95% CI: 0.816-0.974) in the primary cohort and validation cohort, respectively. 447 patients were subdivided into the low-risk group (n=107) and high-risk group (n=340) based on the optimal cut-off value of 21.704. The high-risk patients had a higher likelihood of harboring malignancy.

**Conclusion:** The MRI-based nomogram can be used to achieve an accurate individualized risk prediction of malignancy and reduce unnecessary breast biopsy.

## Introduction

Breast cancer is the most common cancer diagnosed and is the second leading cause of cancer death among women. Breast cancer survival varies substantially by stage at diagnosis, early diagnosis is vital for the prognosis of breast cancer 1. Breast magnetic resonance imaging (MRI) is considered as a crucial imaging tool for diagnosis and preoperative staging of breast cancer 2. And its clinical importance has gradually increased in recent years. Breast MRI has even changed the clinical management for breast cancer.

Besides mammography, ultrasound, positron emission tomography (PET) and MRI are often used as alternate modalities, the advantages of MRI over other modalities are to describe the tumor in dense breast tissue and cancer in a three-dimensional way 3-7. Another advantage of MRI is the high sensitivity (>90%) in breast cancer diagnosis 8. Thus, MRI has the potential to facilitate the diagnosis in patients with breast tumor. However, the specificity of MRI is relatively low. A false-positive result may trigger severe anxiety, unnecessary breast biopsies, and overtreatment. The relatively low specificity of MRI has been attributed to overlapping features in benign and malignant breast tumors 9,10. And the methods of estimating the MRI findings have been somewhat intuitive and relatively inter-observer variability 11. On this account, the American College of Radiology (ACR) issued and updated the Breast Imaging Reporting and Data System (BI-RADS) MRI lexicon which standardized the terminology of morphological features and kinetic curve of breast tumors 12. Even so, BI-RADS didn't identify the correlation between tumor characteristics and malignancy risk. According to the morphological and dynamic contrast-enhanced (DCE) features, the conventional diagnosis of breast tumor on MRI is mainly based on the experience of radiologists. there was not a predictive tool to evaluate the individual risk of malignant tumor based on breast MRI.

In view of these reasons, the following hypothesis immediately puts forward: whether a predictive tool is constructed to evaluate the individual risk of malignancy in patients with breast tumor based on morphological and DCE features?

Recently, nomogram which creates a simple graphical representation of a predictive statistical model 13, has become widely used as predictive tools for diagnosing malignancies. Briefly, A nomogram, which is a user-friendly pictorial representation of mathematical tool, could be applied to predict a numerical probability of an event for an individual patient^14^.

Therefore, the purpose of this study was to establish a preoperative nomogram incorporating morphological and DCE features to individually predict the risk of malignancy in patients with breast tumor.

## Methods

### Study Population

A total of 447 consecutive female patients who were suspected to have solid breast tumor (BI-RADS 4 and 5) on MRI examination were enrolled from March 2015 to January 2018. Our institutional review board approved this retrospective study and waived the requirement for informed consent. The inclusion criteria were as follows: 1) age >20; 2) underwent breast biopsy or surgical excision; 3) underwent MRI examination; The exclusion criteria were as follows: 1) underwent neoadjuvant chemotherapy; 2) non-mass enhancement (NME) or multiple masses. The patients were separated into two cohorts: the primary cohort (n=326) and the validation cohort (n=121).

### MRI Examination

All MRIs were performed with a 1.5 T MRI scanner (Magnetom Avanto; Siemens Medical Solutions, Erlangen, Germany) using an 8-channel breast coil with the patient positioned in the center of the magnet in the prone position. Axial dynamic contrast-enhanced images using a 3D T1-weighted volume interpolated body examination (T1W-VIBE) sequence (TR/TE, 4.6/1.5 msec; flip angle, 10°; FOV, 360×360 mm^2^; matrix, 205 ×256; slice thickness, 2mm and gap, 0 mm) were acquired before and eight dynamic scans after a bolus injection of 0.2mmol/kg Gd-DTPA (Omniscan, GE Healthcare, Ireland) at a rate of 2ml/s, followed by a 20-ml saline flush. In the postprocessing workstation, the region of interest was placed in the enhancement of the largest slice of the tumor to obtain a kinetic curve.

### Image interpretation

According to the BI-RADS 12, morphological and DCE features were independently reviewed by two radiologists (with 8 years and 5 years of experience in breast imaging) without knowledge of the histopathological diagnosis. If the assessments were inconsistent, a consensus was reached by the senior radiologist. The morphological features included tumor size, shape (oval, round or irregular), and margin (circumscribed or non-circumscribed). The DCE features included internal enhancement characteristics (homogeneous or non-homogeneous) and the delayed enhancement features of the kinetic curve (persistent or non-persistent). In addition, age and tumor location were also included.

### Construction of the MRI-based nomogram

Univariate logistic regression analysis was performed to identify the potential MRI features associated with the likelihood of malignancy in patients with breast masses. Subsequently, multivariate logistic regression analysis was conducted to determine the independent predictors. Finally, based on the multivariate logistic regression analysis, the MRI-based nomogram was constructed using the primary cohort, and validated internally by the validation cohort.

### Assessment of MRI-based nomogram performance

The MRI-based nomogram performance was quantified with respect to the discrimination, calibration ability and clinical utility. The Hosmer-Lemeshow test was used to evaluate the goodness-of-fit of the MRI-based nomogram, and the area under the curve (AUC) value of receiver operating characteristic curve (ROC) was carried out to quantify the discrimination ability of the nomogram. Subsequently, a calibration curve was performed to assess the calibration ability of the nomogram. Decision curve analysis was used to evaluate the clinical utility of the model by calculating the net benefits for a range of threshold probabilities.

### Statistical analysis

Univariate and multivariate logistic regression analyses were performed to identify the independent variables. All data analyses were conducted by using Stata/MP, version 13.0 (StataCorp LP, College Station, TX) and R software version 3.4.1. P value <0.05 was considered statistically significant difference.

## Results

### Study Population

Our study population consisted of 447 female patients. The age of included patients ranged from 24 to 78 years, with a mean age of 49 years. The median tumor size was 25mm. Of the 447 breast masses identified in 447 patients, 375 were malignant and 72 were benign. The baseline characteristics of our study were showed in Table 1.

### Predictors of malignancy based on MRI findings

**Figure 1:**
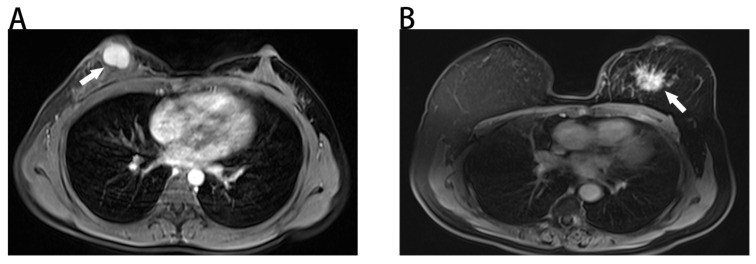
Benign and malignant breast masses on MRI. **(A)** Benign-looking breast mass of 17×12mm (arrow), oval, circumscribed margin and homogeneous enhancement in a 40-year-old woman. Pathological diagnosis: fibroadenoma. **(B)** Malignant-looking breast mass of 36×32mm (arrow), irregular, non-circumscribed margin and heterogeneous enhancement in a 60-year-old woman. Pathological diagnosis: invasive ductal carcinoma.

The results of univariate and multivariate logistic regression analyses were illustrated in Table 2. Based on the result of univariate logistic regression analysis, we found that age, tumor size, mass shape, mass margin, internal enhancement characteristics, and kinetic curve were significantly associated with malignancy. According to the result of multivariate logistic regression analysis, five variables were identified as independent predictors in our study, including age, tumor size, mass margin, internal enhancement characteristics and kinetic curve. Typical MRI findings of benign and malignant breast masses were illustrated in Fig. 1.

### Development of the MRI-based nomogram

**Figure 2:**
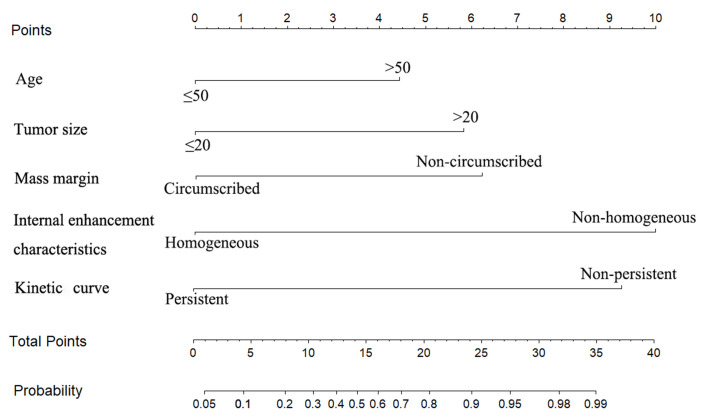
The MRI-based nomogram to predict the risk of malignancy in patients with breast masses based on MRI findings.

The multivariate logistic regression analysis identified age, tumor size, mass margin, internal enhancement characteristics and kinetic curve as independent variables (Table 2). The MRI-based nomogram was constructed and presented in Fig. 2. A total score was calculated using only five parameters of age, tumor size, mass margin, internal enhancement characteristics and kinetic curve. Then, the total score can be applied to point out the frequency of malignant tumors for individual patients.

### Evaluation of the MRI-based nomogram performance

**Figure 3:**
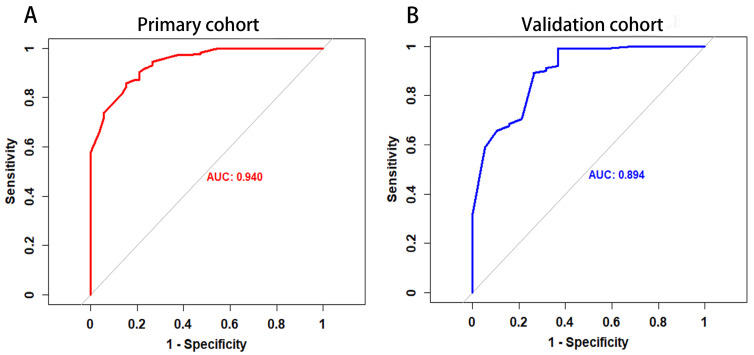
The ROC curve of MRI-based nomogram in the primary cohort and the validation cohort.

**Figure 4:**
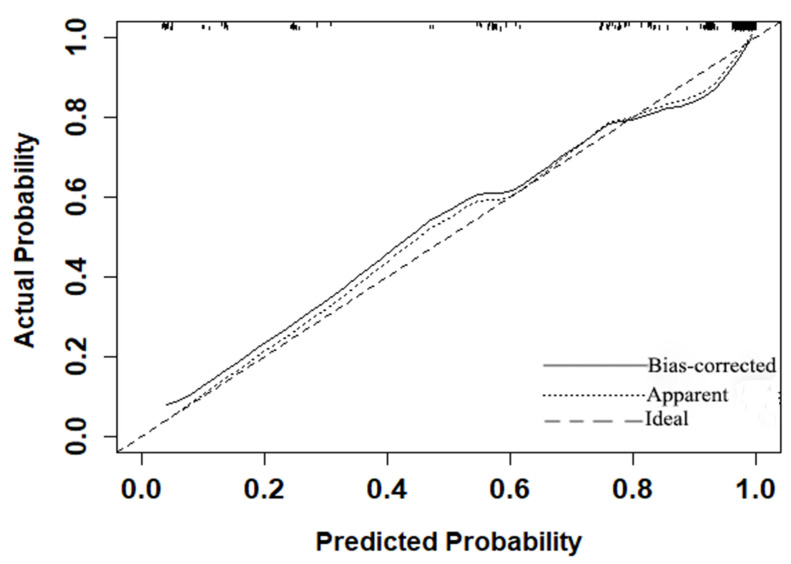
Calibration curve of the MRI-based nomogram to predict the risk of malignancy in patients with breast masses.

The *P* value of the Hosmer-Lemeshow goodness-of-fit test was 0.532, which showed the MRI-based nomogram fitted well. The AUC of the MRI-based nomogram in the primary cohort and the validation cohort were 0.940 (95% CI: 0.911-0.970) and 0.894 (95% CI, 0.816-0.974), which demonstrated the good discrimination ability of our model (Fig. 3). And the calibration curve of the MRI-based nomogram suggested good agreement between prediction and observation (Fig. 4).

MRI-based nomogram scores were assigned for each independent variable in Table 3. To achieve the maximum Youden index (sensitivity+specificity-1) of the total MRI-based nomogram scores, 21.704 was identified as the optimal cutoff value of total nomogram scores in the primary cohort. 447 patients were subdivided into the low-risk group (total nomogram scores ≤21.704, n=107) and high-risk group (total nomogram scores>21.704, n=340) based on the optimal cut-off value. Encouragingly, the high-risk patients had a higher likelihood of harboring malignancy than the low-risk patients (all *P* < 0.001). The sensitivity, specificity, accuracy, false negative rate and false positive rate of the MRI-based nomogram were 93%, 75%, 90%, 7.5% and 25% respectively.

### Clinical utility of MRI-based nomogram

**Figure 5:**
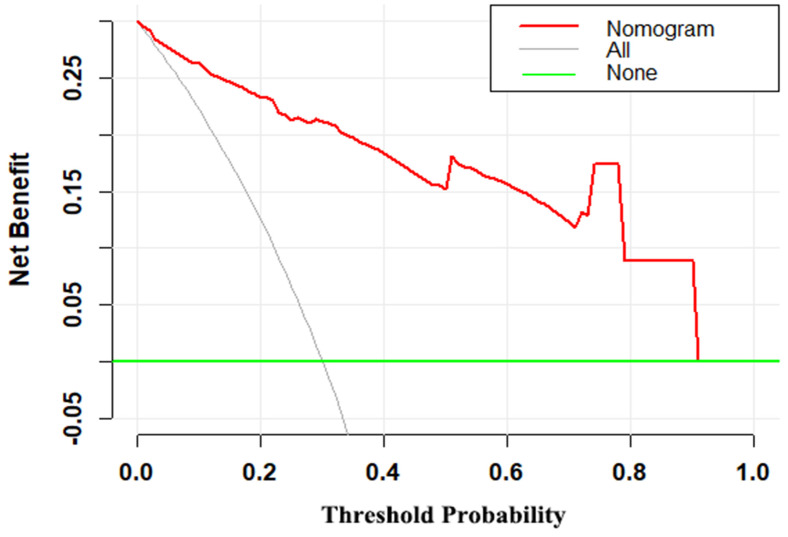
Decision curve for the MRI-based nomogram. The red line represents the MRI-based nomogram. The gray line represents the hypothesis that all patients had malignant mass. The green line represents the hypothesis that all patients had benign mass. The x-axis represents the threshold probability. The y-axis represents the net benefit.

The decision curve demonstrated that the MRI-based nomogram had good clinical utility across the wider range of threshold probability (Fig 5).

## Discussion

In this study, we have first proposed and established a diagnostic MRI-based nomogram which could individually predict the likelihood of malignancy in patients with breast masses. Nomogram has an ability to provide an individual risk of a clinical event for personalized treatment 14. The MRI-based nomogram incorporated patients age, tumor size and three MRI parameters (mass margin, internal enhancement characteristics and kinetic curve). It is generally known that the ROC-AUC of 0.7-0.8 is deemed favorable and the ROC-AUC of 0.81-0.90 is excellent. Thus, the discrimination ability of the MRI-based nomogram was outstanding (ROC-AUC=0.940). Additionally, the Hosmer-Lemeshow goodness-of-fit test yielded the *P* value of 0.532, showing a good fit to the MRI-based nomogram. Besides, the calibration curve demonstrated good calibration ability of the MRI-based nomogram. The decision curve analysis has been recommended to assess the potential clinical usefulness of model 15-20. This important method gains novel insight into clinical consequences based on threshold probability, from which the net benefit can be derived 14,15,21. The decision curve demonstrated that the MRI-based nomogram had better clinical utility with the wider range of threshold probabilities. Furthermore, the MRI-based model also can make effective risk stratification in the diagnosis setting. Therefore, the MRI-based nomogram performed well for preoperative differential diagnosis between benign and malignant breast masses, with favorable clinical utility, risk stratification, discrimination, and calibration ability.

Our study developed a non-invasive preoperative nomogram for predicting the risk of malignancy in patients with breast masses based on MRI findings. Three MRI parameters were adopted as independent indicators to estimate the probability of malignancy in the MRI-based nomogram, including mass margin, internal enhancement characteristics and kinetic curve. Similarly, several prior studies had revealed breast masses with irregular shape, spiculated margin and plateau or washout patterns curve had greater probability of malignancy 11,22-25. Mass shape does appear to be a potential independent variable of malignancy. Nevertheless, in the present study, mass shape was confirmed as an indicator of malignancy in a univariate analysis but lost the statistical significant when added to the multivariate analysis, which was in line with previous study 22. Multiple previous studies have combined the morphologic and DCE features to evaluate the likelihood of malignancy for breast MRI masses based on BI-RADS MRI descriptors 22,23. However, these reports only identified which BI-RADS descriptors were associated with malignancy, cannot provide objective individualized risk prediction in patients with breast mass. Thereby, providing individualized risk prediction by a practical tool remained absence.

The MRI-based nomogram has some clinical significance. First, the MRI-based nomogram only includes five readily available preoperative parameters: age, tumor size, mass margin, internal enhancement characteristics and kinetic curve. Thus, the MRI-based nomogram can easily calculate the risk of malignancy for individual patient. Second, when two radiologists have inconsistent diagnosis of breast lesions, the MRI-based nomogram can be used as a user-friendly tool to reduce inter-observer variability. Third, based on the MRI-based nomogram, breast masses with a low likelihood of malignancy may be qualified for short-interval follow-up rather than breast biopsy. Fourth, the MRI-based nomogram can also provide effective communication among radiologists, oncologists, and patients.

There are some limitations in the study. First, most cases were malignant, but the result was significant. Second, this study was conducted at one institution and was a retrospective study. the multicenter validation studies should be performed before its clinical application in the future. Third, future investigations are needed to establish a preoperative nomogram for predicting the risk of malignancy in patients with non-mass enhancement. Fourth, the initial enhancement feature of the kinetic curve was not included in our study.

## Conclusion

In summary, the proposed MRI-based nomogram, a preoperative prediction tool which incorporates the morphological and DCE features, suggests favorable predictive accuracy for malignancy in patients with breast masses and makes effective risk stratification in the diagnosis setting. The MRI-based nomogram may be used as an important decision-making tool in differentiating malignant from benign breast masses and reducing unnecessary breast biopsy in the low-risk patients.

## Figures and Tables

**Table 1:** Baseline characteristics of the study population

Characteristics	Total cohort	Primary cohort	Validation cohort	*P* value
	No. (%)	No. (%)	No. (%)	
No. of patients	447	326	121	
Age (years)				0.792
mean ± SD*	49.6 ± 10.5	49.3 ± 10.5	50.4 ± 10.6	
≤50	241 (53.9)	177 (54.3)	64 (52.9)	
>50	206 (46.1)	149 (45.7)	57 (47.1)	
Tumor size (mm)				0.413
Median (IQR)	25 (18,34)	25 (18,34)	25 (18,33)	
≤20	160 (35.8)	113 (34.7)	47 (38.8)	
>20	287 (64.2)	213 (65.3)	74 (61.2)	
Tumor location				0.677
UOQ	203 (45.4)	150 (46.0)	53 (43.8)	
Others^a^	244 (54.6)	176 (54.0)	68 (56.2)	
Mass shape				0.444
Oval or round	264 (59.1)	189 (58.0)	75 (62.0)	
Irregular	183 (40.9)	137 (42.0)	46 (38.0)	
Mass margin				0.137
Non-circumscribed^b^	352 (78.7)	251 (77.0)	101 (83.5)	
Circumscribed	95 (21.3)	75 (23.0)	20 (16.5)	
Internal enhancement characteristics				0.175
Non-homogeneous^c^	350 (78.3)	250 (76.7)	100 (82.6)	
Homogeneous	97 (21.7)	76 (23.3)	21 (17.4)	
Kinetics curve				0.550
Non-persistent	400 (89.5)	290 (89.0)	110 (90.9)	
Persistent	47 (10.5)	36 (11.0)	11 (9.1)	
BI-RADS category				0.960
4	206 (46.1)	150 (46.0)	56 (46.3)	
5	241 (53.9)	176 (54.0)	65 (53.7)	
Histopathological type				0.331
Fibroadenoma	38 (8.5)	30 (9.2)	8 (6.6)	
Other benign^d^	34 (7.6)	23 (7.1)	11 (9.1)	
IDC	339 (75.8)	243 (74.5)	96 (79.3)	
Other malignant^e^	36 (8.1)	30 (9.2)	6 (5.0)	
Pathological diagnosis				0.887
Benign	72 (16.1)	53 (16.3)	19 (15.7)	
Malignant	375 (83.9)	273 (83.7)	102 (84.3)	

Note. *Data are means±standard deviation. IQR = Interquartile range, which is the 25th percentile, 75th percentile. UOQ = upper outer quadrant. ^a^Others: UIQ = upper inner quadrant, LIQ = lower inner quadrant, LOQ = lower outer quadrant. ^b^Non-circumscribed: speculated, irregular. ^c^Non-homogeneous: heterogeneous, rim. ^d^Other benign: benign phyllodes tumor 18, papilloma 11, radial scar 5. IDC: invasive ductal carcinoma. ^e^Other malignant: ductal carcinoma in situ 3, papillary carcinoma 6, adenoid cystic carcinoma 2, metaplastic carcinoma 4, mucinous carcinoma 7, invasive lobular carcinoma 14.

**Table 2:** Univariate and multivariate logistic regression analyses in the primary cohort

Variables	Univariate analysis		Multivariate analysis	
	OR (95% CI)	*P* value	OR (95% CI)	*P* value
Age (years)				
≤50	Referent		Referent	
>50	4.460 (2.154-9.236)	<0.001	2.767 (1.046-7.320)	0.040
Tumor size (mm)				
≤20	Referent		Referent	
>20	7.491 (3.847-14.587)	<0.001	3.579 (1.414-9.061)	0.007
Tumor location				
UOQ	Referent			
Others^a^	1.057 (0.586-1.906)	0.853		
Mass shape				
Oval or round	Referent		Referent	
Irregular	7.227 (2.991-17.462)	<0.001	1.414 (0.434-4.603)	0.565
Mass margin				
Circumscribed	Referent		Referent	
Non-circumscribed^b^	11.326 (5.854-21.916)	<0.001	3.547 (1.328-9.470)	0.012
Internal enhancement characteristics				
Non-homogeneous^c^	Referent		Referent	
Homogeneous	0.037 (0.018-0.079)	<0.001	0.116 (0.046-0.292)	<0.001
Kinetics curve				
Persistent	Referent		Referent	
Non-persistent	25.330 (11.044-58.076)	<0.001	7.875 (2.547-24.344)	<0.001

Note. UOQ = upper outer quadrant. ^a^Others: UIQ = upper inner quadrant, LIQ = lower inner quadrant, LOQ = lower outer quadrant. ^b^Non-circumscribed: speculated, irregular. ^c^Non-homogeneous: heterogeneous, rim.

**Table 3:** MRI-based nomogram score for each variable

Variables	Nomogram Score
Age (years)	
≤50	0
>50	4.430
Tumor size (mm)	
≤20	0
>20	5.835
Mass margin	
Circumscribed	0
Non-circumscribed^a^	6.218
Internal enhancement characteristics	
Homogeneous	0
Non-homogeneous^b^	10
Kinetics curve	
Persistent	0
Non-Persistent	9.301
Risk stratification	
Low-risk	≤21.704
High-risk	>21.704

Notes. ^a^Non-circumscribed: speculated, irregular; ^b^Non-homogeneous: heterogeneous, rim

## Abbreviations

DCE: dynamic contrast-enhanced
MRI: magnetic resonance imaging
BI-RADS: Breast Imaging Reporting and Data System
ACR: American College of Radiology
NME: non-mass enhancement
AUC: area under the curve
ROC: receiver operating characteristic curve
